# The Significant Effects of the COVID-19 on Leisure and Hospitality Sectors: Evidence From the Small Businesses in the United States

**DOI:** 10.3389/fpubh.2021.753508

**Published:** 2021-09-27

**Authors:** Zhou Lu, Yunfeng Shang, Linchuang Zhu

**Affiliations:** ^1^School of Economics, Tianjin Univesity of Commerce, Tianjin, China; ^2^School of Hospitality Administration, Zhejiang Yuexiu University, Shaoxing, China

**Keywords:** COVID-19 shocks, leisure and hospitality, accommodation and food services, small businesses, bootstrap unit-root test for a random walk with drift

## Abstract

This paper uses the daily seasonally-adjusted data for net revenues and openings of small businesses in the accommodation, food services, leisure, and hospitality sectors in the United States from January 10, 2020, to June 24, 2021. The results from the Dorta-Sanchez bootstrap unit-root test for a random walk with drift show that the COVID-19 crisis has significantly affected revenues and openings of small leisure and hospitality firms. Moreover, the results remain valid when the data for the national level and 51 states are considered.

## Introduction

The COVID-19 pandemic is one of the largest pandemics in the industrialized world. It has significantly affected all sectors almost in all countries. Since the new type of coronavirus is more lethal and easily contagious than the common flu, governments have had to take many measures to slow the spread of the virus (1). Governments have imposed lockdowns, including closures of accommodation and hospitality facilities, leisure activities, restaurants, and show businesses (2, 3). Governments have also implemented several restrictions on domestic mobility and international travel during the COVID-19 era (4), and this issue has negatively affected the tourism sector (5). The precautionary measures and the widespread use of COVID-19 vaccines have caused significant changes in small business revenues and openings (6).

Understanding the stochastic properties of small business revenues and openings is also essential for macroeconomic variables, such as business cycles, employment, inflation expectations, job openings, and wages (7–9). At this stage, if small-business indicators do not follow a stationary process, this issue indicates an external shock (i.e., the COVID-19 pandemic) that has significantly affected small businesses in the related sector and entrepreneurship behaviors. The evidence of rejecting the stationarity of the business indicators means the significant changes of business cycles (10). Significant changes in business indicators can also affect employment, inflation expectations, job openings, and wages.

Previous papers show the significant effects of uncertainty shocks (e.g., financial crises, natural disasters, political instability, terrorist attacks) on accommodation, food services, leisure and hospitality sectors in the United States (11). There are also previous papers to examine the effects of the COVID-19 crisis on small businesses in the United States. For example, Bartik et al. (12) use the survey data from 5,800 small firms in the United States from March 28, 2020, to April 4, 2020. The authors find the significant impact of the COVID-19 crisis on small businesses, particularly financially fragile firms. The impact quickly transmits (within a few weeks), and firm closure is negatively related to the expectations, which are heterogeneous based on the length of the COVID-19 crisis. The authors also compare the effectiveness of loan reliefs with grants-based stimulus programs. Fairlie and Fossen (13) observe that sales losses in California during the 2020Q2 were greatest in accommodations, arts, entertainment, recreation, and restaurants. Huang et al. (14) find that business closures cause around 30% decline to the non-salaried workers' employment in entertainment, food, hospitality, and leisure sectors in the United States between March 2020 and April 2020. Khan et al. (15) use the leisure sector employment data in the United States from February 1, 2020, to July 31, 2020. The authors find that museums, performing arts, and sports have been the worst-affected businesses during the COVID-19 era.

Given this backdrop, this paper analyzes the validity of the hypothesis of whether the COVID-19 crisis has significantly affected revenues and openings of businesses in the accommodation, food services, leisure, and hospitality sectors in the United States. Our main hypothesis is to reject the stationarity of the revenues and openings of small leisure and hospitality firms. To test the main hypothesis, we consider the daily seasonally-adjusted data, introduced by Chetty et al. (6), for net revenues and openings of small businesses in accommodation, food services, leisure and hospitality sectors in the United States from January 10, 2020, to June 24, 2021. At this stage, we consider the data at the national and state levels. For this purpose, we utilize the bootstrap unit-root test for a random walk with the drift of Dorta and Sanchez (16). The bootstrapped critical values decrease the size distortions following the bootstrap procedure in Park (17).

To the best of our knowledge, this paper provides the first empirical evidence using the daily seasonally-adjusted data for net revenues and openings of small businesses in Chetty et al. (6) for accommodation, food services, leisure, and hospitality sectors in the United States. For this purpose, we aim to examine the dynamics of small businesses in the leisure and hospitality sector in the United States during the COVID-19 period. Moreover, we utilize the Dorta-Sanchez bootstrap unit-root test for a random walk with drift to address poor sample size in small business data. Therefore, we aim to reduce the shortcomings of traditional unit-root tests. As a result, we find that the COVID-19 crisis has significantly affected the revenues and openings of small leisure and hospitality firms in the United States. Moreover, this result is valid when the data for the national level and 51 states are considered.

The remainder of the study is organized as follows. Section 2 clarifies the details of the dataset and the Dorta-Sanchez bootstrap unit-root test methodology. Section 3 presents the empirical results. Section 4 concludes.

## Dataset and Test Methodology

### Dataset

This paper uses the seasonally-adjusted data for net revenues and openings of small businesses in accommodation, food services, leisure and hospitality sectors in the United States from January 10, 2020, to June 24, 2021. The frequency of the data is daily, and the sample is based on the data availability. Note the first case of the COVID-19 in the United States is recorded on January 20, 2020. Therefore, our dataset captures the COVID-19 era. Both series are defined as the relative change between the given date and the average from January 4, 2020, to January 31, 2020. These series are proposed by Chetty et al. (6) at https://tracktherecovery.org/, and the data are provided by Womply (a private-sector firm in the United States). The dataset in Chetty et al. (6) has been used by various empirical papers related to the COVID-19 pandemic [see, e.g., (18, 19)].

**Figure 1:**
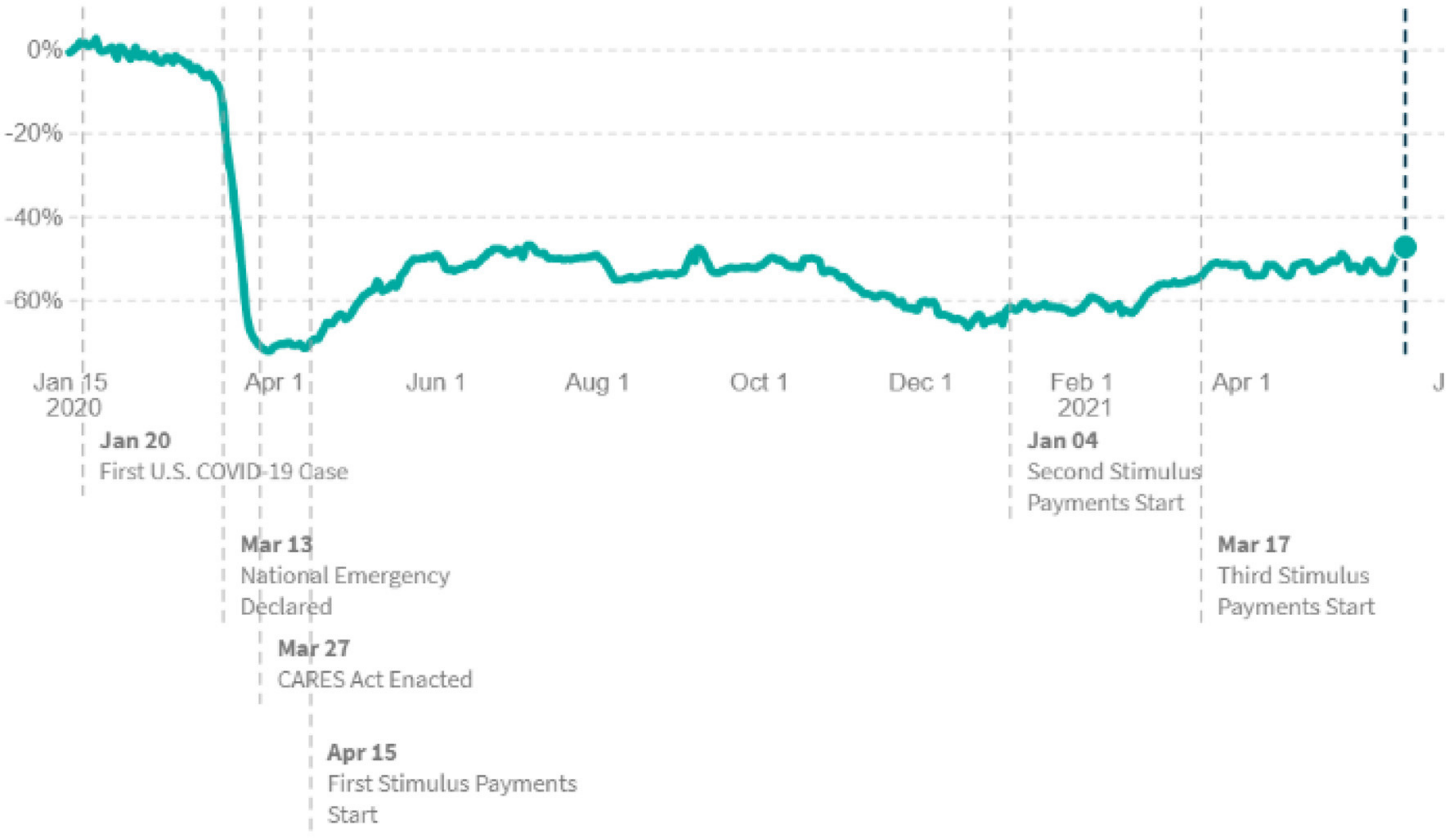
Small businesses net revenues: leisure and hospitality sector (national level, % of change). Source: https://tracktherecovery.org/ proposed by Chetty et al. (6).

According to the data in Figure 1, as of June 24, 2021, net small businesses revenues in the leisure and hospitality sector in the United States reduced by 47.3% compared to January 2020.

### Dorta-Sanchez Bootstrap Unit-Root Test Methodology

We utilize the bootstrap unit-root test for a random walk with drift introduced by Dorta and Sanchez (16). The Dorta-Sanchez unit-root test corrects the possible bias in the data generation process (DGP) that corresponds to a random walk with a non-zero drift for small and medium sample sizes. For example, Hylleberg and Mizon (20) show the poor sample size in the Augmented Dickey-Fuller (ADF) test in the small number of observations. Hamilton (21) suggests the ordinary least squares (OLS) estimation with the standard t and F distributions to decrease the sample bias. Park (17) uses the ADF unit-root test for autoregressive (AR) unit-root models with bootstrapped critical values to address possible sample bias. At this stage, Dorta and Sanchez (16) calculate the bootstrapped critical values for the unit-root test methodology of Park (17). Poor sample size can also be an issue in our case, given that there are sub-periods in the sample during the COVID-19 period (See Figure 1).

The null hypothesis of the Dorta-Sanchez unit-root test is as follows*H*_*o*_ : δ = 0 The model can be defined as follows:


(1) $$\begin{array}{l} \Delta y_{t}=\alpha +\delta y_{t-1}+\overset{p}{\underset{i=1}{\sum }}\beta \Delta y_{t-i}+\varepsilon_{t} \end{array}$$

ε_*t*_is the independent and identically distributed (iid) error term. The fitted regression can be written as follows:


(2) $$\begin{array}{l} \Delta y_{t}=\alpha +\overset{p}{\underset{i=1}{\sum }}\beta \Delta y_{t-i}+\varepsilon_{t} \end{array}$$

Park (17) shows that resampling the restricted model in Eq. (2) will be better than the unrestricted model in Eq. (1). Following the findings in Park (17), estimated residuals (${\hat{\varepsilon }}_{t}$) based on the bootstrap sample sizes can be calculated. The new residuals () with the bootstrap method can be written as such:


(3) $$\begin{array}{l} \left({\hat{\varepsilon }}_{t}-\frac{1}{n}\overset{n}{\underset{i=1}{\sum }}{\hat{\varepsilon }}_{i}\right) \end{array}$$

*t* = *1;…; n*

For each bootstrap sample ($y_{t}^{*}$), the fitted regressions can be written as such:


(4) $$\Delta y_{t}^{*}=\alpha^{*}+\delta^{*}y_{t-1}^{*}+\sum_{i=1}^{p}\beta_{i}^{*}\Delta y_{t-i}^{*}+v_{t}$$

The original sample based on fitted regression is as follows:


(5) $$\begin{array}{l} \Delta y_{t}=\mu +\delta y_{t-1}+\overset{p}{\underset{i=1}{\sum }}\beta_{i}\Delta y_{t-i}+\varepsilon_{t} \end{array}$$

The t statistic for δ is calculated, compared to the bootstrapped critical values, which are defined above. If the t statistic is lower than the bootstrapped critical values, the unit root hypothesis will be rejected. Furthermore, the *p*-values on comparing bootstrapped critical values and t statistics are also provided (16). Finally, the optimal number of lags is also determined by the Akaike Information Criteria (AIC).

If we obtain stationary series, we can calculate the Half-life (HL) values to detect how many days COVID-19 shocks survive. We calculate the HL values, as such:


(6) $$\begin{array}{l} Half-life=|\text{ln }\left(0.5\right)|/|\text{ln }\left(\rho \right)| \end{array}$$

In Equation 6, ρ is the AR coefficient in AR (1) process *Y*_*t*_ = ρ*Y*_*t*−1_ + ε_*t*_.

## Empirical Results

Table 1 reports the findings of the bootstrap unit-root test for a random walk with drift proposed by Dorta and Sanchez (16) at the national level for small businesses revenues and openings of two sectors: (i) leisure and hospitality and (ii) accommodation and food services. The results indicate that small businesses revenues and openings in two sectors follow the random walk with drift process. In other words, the stationarity of the small business indicators is rejected. Moreover, these results are robust to different lag selection criteria.

**Table 1:** Results of the bootstrap unit-root test for a random walk with drift (small businesses revenues and openings in different sectors, national level).

**Small Businesses Revenues (Leisure and Hospitality)**				
Criteria & (Lag)	Test Stat.	Prob.	5% CVs	HL (Days)
AIC (1)	−2.479	(0.092)	−2.799	–
**Small Businesses Revenues (Accommodation and Food Services)**				
Criteria & (Lag)	Test Stat.	Prob.	5% CVs	HL (Days)
AIC (1)	−2.458	(0.093)	−2.783	–
**Small Businesses Openings (Leisure and Hospitality)**				
Criteria & (Lag)	Test Stat.	Prob.	5% CVs	HL (Days)
AIC (1)	−2.299	(0.090)	−2.566	–
**Small Businesses Openings (Accommodation and Food Services)**				
Criteria & (Lag)	Test Stat.	Prob.	5% CVs	HL (Days)
AIC (1)	−2.258	(0.066)	−2.420	–

*CVs: Bootstrapped Critical Values. The CVs are obtained by 500 bootstrap replicates with 530 observations. For details, refer to Dorta and Sanchez (16). The optimal number of lags is selected by the AIC. Null hypothesis: random walk with drift; Alternative hypothesis: series are stationary*.

Tables 2, 3 report the bootstrap unit-root test results for a random walk with drift proposed by Dorta and Sanchez (16) for the state level for small businesses' revenues and openings for leisure and hospitality. The results of the Dorta-Sanchez test in Table 2 show that nearly all small businesses revenues series follow the random walk with drift process. The only exception is observed in Alaska, and the HL of the COVID-19 shock is 64 days.

**Table 2:** Results of the bootstrap unit-root test for a random walk with drift (small businesses revenues, leisure and hospitality).

**State**	**Test stat**.	**Prob**.	**5% CVs**	**HL (Days)**
AL	−2.188	(0.238)	−2.911	–
AK	−2.849^**^	(0.032)	−2.849	64
AZ	−2.673	(0.076)	−2.833	–
AR	−2.044	(0.300)	−2.885	–
CA	−2.533	(0.060)	−2.559	–
CO	−2.461	(0.106)	−2.806	–
CT	−2.532	(0.088)	−2.713	–
DE	−2.465	(0.118)	−2.961	–
DC	−2.324	(0.144)	−2.483	–
FL	−2.562	(0.116)	−3.099	–
GA	−2.006	(0.216)	−2.714	–
HI	−2.320	(0.136)	−2.831	–
ID	−1.943	(0.346)	−3.023	–
IL	−2.237	(0.140)	−2.801	–
IN	−2.407	(0.102)	−2.838	–
IA	−1.850	(0.398)	−2.886	–
KS	−2.327	(0.158)	−2.899	–
KY	−2.181	(0.260)	−3.006	–
LA	−2.402	(0.112)	−2.786	–
ME	−2.360	(0.150)	−2.835	–
MD	−2.023	(0.200)	−2.769	–
MA	−2.573	(0.068)	−2.668	–
MI	−2.040	(0.194)	−2.850	–
MN	−2.267	(0.195)	−2.888	–
MS	−2.045	(0.190)	−2.669	–
MO	−2.144	(0.316)	−3.038	–
MT	−1.698	(0.608)	−3.121	–
NE	−1.874	(0.344)	−2.856	–
NV	−1.772	(0.540)	−3.192	–
NH	−2.213	(0.234)	−2.929	–
NJ	−2.541	(0.060)	−2.602	–
NM	−1.979	(0.184)	−2.691	–
NY	−2.417	(0.064)	−2.506	–
NC	−2.366	(0.160)	−2.878	–
ND	−2.268	(0.194)	−2.871	–
OH	−2.426	(0.148)	−2.856	–
OK	−2.110	(0.206)	−2.795	–
OR	−2.443	(0.134)	−2.757	–
PA	−2.446	(0.092)	−2.777	–
RI	−2.346	(0.108)	−2.717	–
SC	−2.113	(0.228)	−2.812	–
SD	−2.103	(0.276)	−2.892	–
TN	−2.016	(0.386)	−2.965	–
TX	−2.393	(0.096)	−2.659	–
UT	−2.705	(0.078)	−2.851	–
VT	−2.418	(0.110)	−2.801	–
VA	−2.239	(0.120)	−2.668	–
WA	−2.235	(0.252)	−3.023	–
WV	−2.368	(0.138)	−2.736	–
WI	−1.938	(0.334)	−2.911	–
WY	−1.954	(0.340)	−3.028	–

*CV: Bootstrapped Critical Value. The CVs are obtained by 500 bootstrap replicates with 530 observations. For details, refer to Dorta and Sanchez (16). The optimal number of lags is selected by the AIC. Null hypothesis: random walk with drift; Alternative hypothesis: series are stationary*.

** *p < 0.01*.

**Table 3:** Results of the bootstrap unit-root test for a random walk with drift (small businesses openings, leisure and hospitality).

**State**	**Test stat**.	**Prob**.	**5% CVs**	**HL (Days)**
AL	−2.303	(0.102)	−2.619	–
AK	−2.613^**^	(0.030)	−2.411	92
AZ	−2.182	(0.120)	−2.619	–
AR	−2.110	(0.212)	−2.885	–
CA	−2.367	(0.064)	−2.524	–
CO	−2.379	(0.108)	−2.716	–
CT	−2.244	(0.096)	−2.574	–
DE	−2.167	(0.123)	−2.452	–
DC	−2.213	(0.088)	−2.450	–
FL	−2.353	(0.086)	−2.474	–
GA	−1.906	(0.118)	−2.291	–
HI	−2.285	(0.112)	−2.703	–
ID	−1.730	(0.310)	−2.698	–
IL	−2.272	(0.110)	−2.703	–
IN	−2.263	(0.112)	−2.544	–
IA	−1.972	(0.226)	−2.913	–
KS	−2.282	(0.098)	−2.731	–
KY	−2.401	(0.096)	−2.829	–
LA	−2.289	(0.128)	−2.690	–
ME	−2.453	(0.084)	−2.676	–
MD	−2.107	(0.108)	−2.497	–
MA	−2.202	(0.062)	−2.338	–
MI	−2.292	(0.078)	−2.513	–
MN	−2.181	(0.140)	−2.863	–
MS	−2.441	(0.124)	−2.683	–
MO	−2.060	(0.196)	−2.717	–
MT	−1.762	(0.260)	−2.633	–
NE	−1.377	(0.378)	−2.845	–
NV	−2.665	(0.075)	−2.879	–
NH	−2.477	(0.080)	−2.806	–
NJ	−2.356	(0.0600)	−2.449	–
NM	−1.998	(0.128)	−2.361	–
NY	−2.417	(0.054)	−2.462	–
NC	−2.565	(0.067)	−2.689	–
ND	−2.134	(0.158)	−2.733	–
OH	−2.227	(0.130)	−2.780	–
OK	−2.041	(0.168)	−2.697	–
OR	−2.263	(0.132)	−2.887	–
PA	−2.317	(0.116)	−2.808	–
RI	−2.226	(0.132)	−2.714	–
SC	−1.878	(0.138)	−2.558	–
SD	−1.552	(0.276)	−2.703	–
TN	−2.367	(0.092)	−2.624	–
TX	−2.158	(0.093)	−2.455	–
UT	−2.234	(0.130)	−2.854	–
VT	−2.185	(0.180)	−2.794	–
VA	−1.938	(0.138)	−2.492	–
WA	−2.267	(0.130)	−2.723	–
WV	−2.221	(0.110)	−2.715	–
WI	−2.384	(0.102)	−2.690	–
WY	−1.876	(0.252)	−2.755	–

*CV: Bootstrapped Critical Values. The CVs are obtained by 500 bootstrap replicates with 530 observations. For details, refer to Dorta and Sanchez (16). The optimal number of lags is selected by the AIC. Null hypothesis: random walk with drift; Alternative hypothesis: series are stationary*.

** *p < 0.01*.

Similarly, the results of the Dorta-Sanchez test in Table 3 indicate that nearly all small businesses openings series follow the random walk with drift process. Again, the only exception is observed in Alaska, and the HL of the COVID-19 shock is 92 days.

In short, we observe that the COVID-19 crisis has significantly affected small businesses' revenues and openings in the United States. Moreover, this evidence is valid when we consider the data at the national and state levels. Regarding the COVID-19 era, our results align with the previous results of Bartik et al. (12, 14, 15) in the United States. Furthermore, we have enhanced these results by using the daily data with the recent unit-root test.

## Conclusion

This paper uses the dataset in Chetty et al. (6) at https://tracktherecovery.org/. It focuses on the small businesses' revenues and openings in accommodation, food services, leisure and hospitality sectors in the United States. We consider the daily data from January 10, 2020, to June 24, 2021. We utilize the recent bootstrap unit-root test for a random walk with drift proposed by Dorta and Sanchez (16). We observe that the COVID-19 crisis has significantly affected the revenues and openings of small leisure and hospitality firms in the United States. The findings are valid when we use data for the national level and 51 states.

Regarding policy implications, the findings show that an external shock, such as the COVID-19 pandemic, has permanently affected the revenues and the openings of leisure and hospitality firms in the United States. Leisure and hospitality firms in all states have performed relatively weak during the COVID-19 era. Rejecting the stationarity in the small business indicators of leisure and hospitality is a strong signal of the business cycles during the COVID-19 era. The significant change during the COVID-19 can be related to declining demand for leisure and hospitality services due to the lockdowns or other limitations on mobility. There are also supply chains problems and restrictions on the supply side of leisure and hospitality firms due to the COVID-19 pandemic.

Our results are consistent with the idea that the COVID-19 pandemic has not affected coastal and inland areas differently. In other words, we find that the COVID-19 pandemic has significantly affected the leisure and hospitality firms in all states. At this stage, implications for supporting struggling small business firms are important to mitigate the devastating effects of the COVID-19 crisis. For instance, the American Rescue Plan in March 2021 provides credit expansion, direct capital injection, and tax reliefs to boost business activities, including the leisure and hospitality sector. Finally, future papers can focus on the other sectors to examine whether the COVID-19 has disproportionately affected small businesses across the United States.

## Data Availability Statement

Publicly available datasets were analyzed in this study. This data can be found here: https://tracktherecovery.org/.

## Author Contributions

ZL: empirical analyses, writing the paper, and supervision. YS: methodology and writing the paper. LZ: data collection and writing the paper. All authors contributed to the article and approved the submitted version.

## Funding

The authors acknowledge the funding from the Philosophy & Social Science Fund of Tianjin City, China. Award #: TJYJ20-012 (Prompting the Market Power of Tianjin City's E-commerce Firms in Belt & Road Countries: A Home Market Effect Approach).

## Conflict of Interest

The authors declare that the research was conducted in the absence of any commercial or financial relationships that could be construed as a potential conflict of interest.

## Publisher's Note

All claims expressed in this article are solely those of the authors and do not necessarily represent those of their affiliated organizations, or those of the publisher, the editors and the reviewers. Any product that may be evaluated in this article, or claim that may be made by its manufacturer, is not guaranteed or endorsed by the publisher.
